# Repeated Low‐Level Inflammatory Challenge Leads to Alterations in the TNF‐CXCL10 Signalling Pathway in Mouse Cerebral Endothelial Cells In Vitro

**DOI:** 10.1111/jnc.70130

**Published:** 2025-06-16

**Authors:** Megan Ritson, Dong Xia, Caroline Wheeler‐Jones, Helen B. Stolp

**Affiliations:** ^1^ Department of Comparative Biomedical Sciences Royal Veterinary College London UK

**Keywords:** apoptosis, cerebrovascular, endothelium, inflammation, proliferation

## Abstract

The mechanism by which chronic systemic inflammation contributes to cerebral endothelial dysfunction and neurological disorders is unclear, although endothelial inflammatory signalling is considered a cornerstone of this process. Here, we have performed transcriptomic analysis of published RNASeq datasets and identified consistent upregulation of the Tumour Necrosis Factor—C‐X‐C Motif Chemokine Ligand 10 (TNF‐CXCL10) signalling pathway in mouse cerebral endothelial cells following a single inflammatory challenge. We subsequently investigated the effects of repeated low‐level inflammation on the modulation of this pathway in a mouse cerebral endothelial cell line, analysing the effect on markers of endothelial cell activation and changes in cellular function, as a potential mechanism underlying the cerebrovascular response to low‐level systemic inflammation. Mouse cerebral endothelial cells (bEnd.3) were exposed to hour‐long treatments with phosphate buffered saline (PBS), a single low concentration of TNF (0.5 ng/mL), repeated low‐concentration TNF (0.5 ng/mL, 1 h × 4 days) or a single cumulative concentration of TNF (2.0 ng/mL). RNA and protein were extracted 4 and 24 h after the final treatment for analysis of gene/protein expression using qRT‐PCR and western blotting. Repeated inflammatory challenge significantly upregulated both Intercellular Adhesion Molecule 1 (ICAM1) and CXCL10 at the mRNA and protein levels. Signal transducer and activator of transcription 1 (STAT1) and phosphorylated‐STAT1 (pSTAT1) protein levels were also increased at 4 and 24 h. Differentially, tumor necrosis factor receptor‐associated factor 2 (TRAF2) and Interferon gamma (IFNγ) gene expression were decreased at 4 h, returning to control levels at 24 h. Functional analysis revealed significant increases in endothelial cell proliferation and apoptosis in the presence of repeated TNF exposure. CXCL10 knockdown with small interfering RNA (siRNA) reduced mean caspase 3/7 activity induced by the repeated inflammatory paradigm. These data suggest an upregulation of the TNF‐CXCL10 pathway in response to low‐level repetitive inflammation in mouse cerebral endothelial cells. Modulation of this pathway may represent a broad therapeutic target for neurovascular disease.

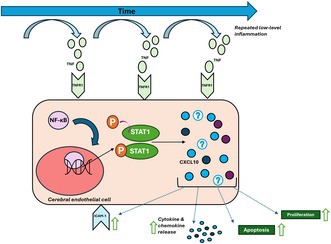

## Graphical abstract

Intermittent, mild pro‐inflammatory systemic disease contributes to cerebrovascular pathology, however the mechanism of low‐level exposure causing injury remains unclear. Our RNA‐Seq analysis reveals continuous activation of tumour necrosis factor (TNF)‐Nuclear Factor kappa B (NF‐κB) signalling and multiple downstream inflammatory mediators in mouse cerebral endothelial cells across various injury models. In vitro studies utilising a model of repeated low‐level inflammation in a mouse brain endothelial cell line show upregulation of the TNF‐ CXCL10 pathway, accompanied by altered endothelial function. Modulating this pathway may provide a broad‐spectrum therapeutic target for cerebrovascular disease.

**Abbreviations**
ATCC: American Type Culture Collection
CXCL10: C‐X‐C Motif Chemokine Ligand 10
CXCR3: C‐X‐C Motif Chemokine Receptor 3
DMEM: Dulbecco's Modified Eagle Medium
EGR1: early growth response factor 1
EGSEA: ensemble of gene set enrichment analysis
ELISA: enzyme‐linked immunosorbent assay
FBS: foetal bovine serum
FDR: false discovery rate
GAPDH: glyceraldehyde‐3‐phosphate dehydrogenase
GEO: Gene Expression Omnibus
HUVEC: human umbilical vein endothelial cells
ICAM1: intercellular adhesion molecule 1
IFNγ: interferon gamma
JAK2: Janus kinase 2
Log2FC: log fold‐change
LPS: lipopolysaccharide
MCAO: middle cerebral artery occlusion
NF‐κB: nuclear factor‐κB
PBS: phosphate buffered saline
qRT‐PCR: quantitative reverse transcription polymerase chain reaction
RNA: ribonucleic acid
siRNA: small interfering RNA
STAT1: signal transducer and activator of transcription 1
TBST: Tris‐Buffered Saline with Tween 20
TNF: tumour necrosis factor
TRAF2: TNF receptor‐associated factor 2

## 1 Introduction

Cerebrovascular disease and cerebrovascular dysfunction are catch‐all terms referring to conditions that affect blood flow to the brain and, as a result, neurological function. These most commonly include stroke or arteriovenous malformations but are increasingly being used to refer to altered vascular function in neurological diseases such as multiple sclerosis, vascular dementia, Alzheimer's and Parkinson's disease (Hatate et al. 2016; Marshall et al. 2016; Shabir et al. 2018). Inflammation and inflammatory mediators are strongly implicated in the altered response of the cerebrovasculature in these conditions (Grammas 2000; Fang et al. 2023; Zhang et al. 2023), and inflammation is now considered a driving force behind the aetiology and progression of cerebrovascular disease (Ritson et al. 2024).

Chronic systemic inflammation, associated with diseases such as atherosclerosis and diabetes, leads to impaired blood–brain barrier integrity resulting from altered structural and functional properties of the endothelium, alongside a marked increase in pro‐inflammatory mediator production and cerebral endothelial activation (Sheikh et al. 2022). This suggests that continuous systemic inflammation across a lifespan may produce incremental endothelial injury, leading over time to cerebrovascular dysfunction. It is also possible that repeated exposure to low‐level inflammation primes the cerebrovasculature, a phenomenon that has been well described for immune cells (Furuhashi et al. 2017), increasing susceptibility to subsequent injury (Stolp and Solito 2024). To date, the specific cerebral endothelial cell responses to systemic inflammation have not been determined.

It is well established that exposure of endothelial cells to inflammatory cytokines in vitro causes activation of downstream proinflammatory signalling pathways, including nuclear factor‐κB (NF‐κB), that negatively affects endothelial homeostasis, impacting essential processes linked to maintaining brain vessel health, including cell survival, angiogenic potential and paracellular tight junction permeability (Giraudo et al. 1998; Sainson et al. 2008; Rastogi et al. 2012; Sawant et al. 2013; Lo et al. 2014; Miyazaki et al. 2017; Zhou et al. 2017; Wang et al. 2021). However, studies have often utilised a high concentration, ‘one‐hit’ model of inflammatory challenge, where the concentration range of the inflammatory agent (e.g., tumour necrosis factor, TNF) used is ~100 fold higher (e.g., 5–20 ng/mL) than levels present in the serum of individuals with neurological disorders such as Parkinson's disease (which vary between 2.5 and 250 pg/mL) (Fu et al. 2023; Wang et al. 2023) or in systemic diseases such as diabetes (varies between 4.5 and 115 pg/mL) (Singhai et al. 2012). Thus, the in vitro experimental conditions used to date do not accurately recapitulate the environment experienced by the cerebral endothelium and therefore misrepresent the vascular effects of ongoing systemic inflammation known to be present in neurovascular pathologies (Sankowski et al. 2015). Given that cytokine levels fluctuate in both neurological and systemic diseases, there is need to better model these variations, providing a more physiologically relevant insight into mechanisms of disease.

In this study, we used transcriptomic data sets from mouse cerebral endothelial cells to identify genes and signaling pathways consistently altered across different injury models and timeframes. The TNF‐C‐X‐C motif chemokine ligand 10 (CXCL10) signaling pathway was identified as a consistent and acutely activated pathway, which was then investigated in a novel proof‐of‐concept in vitro model of repeated low concentration TNF exposure in a mouse cerebral endothelial cell line. Prolonged upregulation of mediators in this pathway, such as TNF, STAT1 and CXCL10, were identified along with functional changes within the cerebral endothelial cells, highlighting the need for further investigation into the mechanisms of cerebrovascular injury and disease risk factors.

## 2 Materials and Methods

### 2.1 RNA‐Seq Data Analysis

Bulk RNA‐seq datasets were selected from the Gene Expression Omnibus (Barrett et al. 2013) and NCBI Sequence Read Archive (Leinonen et al. 2011) using a literature search from a combination of keywords: ‘transcriptomic’, ‘endothelial’, ‘inflammation’, ‘RNA‐Seq’, ‘vasculature’ and ‘injury’. Six comparisons and controls from 3 studies (Munji et al. 2019; Jambusaria et al. 2020; Kodali et al. 2021) were selected to be processed for analysis based on cell type, disease model and data availability (Table S1). Data processing was performed using the Galaxy public server at usegalaxy.eu (v. 1.20.0) (Afgan et al. 2016) alongside R version 4.4.1 (R Core Team 2023).

The Ensemble of Gene Set Enrichment Analysis (EGSEA) (Alhamdoosh et al. 2017) parameters were set to include the top 15 gene sets covering categories such as Hallmark (H), Curated (C2), Immunologic (C7), GenesetDB pathways, disease/phenotype, gene regulation, Gene Ontology (GO) and KEGG gene sets. The false discovery rate (FDR) adjusted *p*‐values of all gene sets were calculated. For an overview of altered pathways/processes, the log fold‐change (Log2FC) of significantly altered gene sets (FDR < 0.05) was recorded. Gene sets with insignificant enrichment (FDR ≥ 0.05) were excluded from further analysis. To enable comparisons across studies, ggplot2 and ggdendro packages were used to create hierarchical clustering and heatmaps using the Log2FC values for the altered gene sets for each comparison. For analysis of individual genes, the Log2FC was calculated for all significantly altered genes (FDR < 0.05) within each identified gene set. Genes with significant changes in expression in at least 4 of the 6 comparisons were then plotted.

STRING (v. 11.5) and Cytoscape (v. 3.9.1) were used to generate a protein–protein interaction network of identified genes of interest. The parameters were set to include 20 first shell interactors and 5 s shell interactors, and the minimum confidence interval was taken to be 0.4. Genes with no interactions were not displayed. The EnrichR platform was used to identify common human transcription factors between the proteins in the network using ChIP Enrichment Analysis (ChEA), ranked by combination score (Kuleshov et al. 2016; Xie et al. 2021).

### 2.2 Culture of bEnd.3 Cells

The immortalised mouse brain endothelial cell line, bEnd.3 (American Type Culture Collection, ATCC, Manassas VA; RRID: CVCL_0170), was grown in complete medium, comprised of Dulbecco's Modified Eagle Medium containing 4 mM L‐glutamine, 4500 mg/L D‐glucose, 1 mM sodium pyruvate, and 1500 mg/L sodium bicarbonate (DMEM; ATCC; cat. no #30‐2002), supplemented with 10% foetal bovine serum (FBS; ATCC; cat. no #30‐2020) and 2% penicillin/streptomycin (GIBCO; cat. no #15140122). Cells were maintained at 37°C and 5% CO_2_ and incubated under these conditions for all experiments.

### 2.3 Cell Treatments

bEnd.3 cells were used between passages 2 and 12. For concentration‐response experiments, cells were seeded in 12‐well plates at a density of 3 × 10^5^ cells per well. Cells were treated with recombinant mouse TNF protein (R&D Systems; cat.no #410‐MT‐010) diluted in phosphate buffered saline (PBS; ThermoFisher; cat.no #10010023), with fresh complete medium added after a 1 h incubation, and cells collected for RNA extraction after 4 h. The data produced were used to determine the range of TNF concentrations for use in subsequent experiments (Figure S1). The effects of exogenous CXCL10, a pro‐inflammatory chemokine, were determined by exposing cells to recombinant mouse CXCL10 (R&D Systems; cat. no #466‐CR) or vehicle alone (PBS) for 24 h.

For experiments involving repeated inflammatory challenge, cells were plated in 6‐ or 12‐well plates at a seeding density of 1 × 10^5^ or 5 × 10^4^ cells per well, respectively. Control groups were treated with PBS for three consecutive days and then exposed to either PBS (negative control), 0.5 ng/mL TNF (single sub‐threshold concentration) or 2.0 ng/mL TNF (positive control) on Day 4. The repeated exposure group received TNF at 0.5 ng/mL on four consecutive days (Figure S2). For each treatment, cells were incubated in PBS or TNF for 1 h, before a complete medium change to remove the exogenous stimulus. Analysis of inflammatory gene and protein responses was then carried out either 4 or 24 h post final treatment, based on findings from the RNA‐Seq analysis. Functional assays were all carried out at the 24‐h time point to allow time for changes to occur, while also aligning with the secondary timepoint of gene/protein analysis.

### 2.4 CXCL10 Knockdown Using Small Interfering RNA

Targeted small interfering RNA (siRNA) was used to knock down CXCL10 expression in bEnd.3 cells. Cells were seeded onto 12‐well plates, and, when ~40% confluent, the medium was removed and OptiMEM medium added. For siRNA optimization, 50 nM of 4 different CXCL10 targeting siRNAs (Dharmacon; cat. no #MQ‐042605‐00‐0002; Table S2) and a scrambled non‐targeting control were prepared and mixed with 6% transfection reagent (Escort III; Merck; cat. no#L3037) made up in OptiMEM (ThermoFisher; cat. no #31985062). Constructs were added dropwise to cells (100 μL per well) and incubated for 4 h, before fresh medium was added and cells incubated for a further 24 h.

Knockdown of CXCL10 using the 4 sequences was assessed by measuring CXCL10 mRNA expression at 24, 48 and 72 h by qRT‐PCR. Evaluation of the 4 siRNAs identified siRNA 2 as the most effective with ~90% knockdown after 72 h, compared to between zero and 40% for the other siRNAs (Figure S4a). Knockdown of CXCL10 at the mRNA and protein levels was then confirmed at the same time points in the presence of TNF (Figure S4b,c).

For knockdown of CXCL10 in repeated TNF exposure experiments, cells were seeded (5 × 10^4^ per well) onto 12‐well plates and transfected with CXCL10 siRNA 2 the following day when they were approximately 40% confluent. Six hours after transfection, cells were treated with either TNF (0.5 ng/mL) or PBS and then treated as described above every 24 h for four consecutive days. Total RNA or protein was extracted 4 h after the final treatment. Functional assays (migration, proliferation, tube formation) were carried out at 24 h post final treatment.

### 2.5 Quantitative Real‐Time Polymerase Chain Reaction (qRT‐PCR)

Total RNA was extracted using the RNeasy kit (Qiagen; cat. no #74104) according to the manufacturer's instructions. RNA concentration was determined using a Nanodrop 1000 spectrophotometer (ThermoFisher Scientific, ND1000). The qPCRBio Probe 1‐step Go HiROX kit (PCR Biosystems; cat. no PB25.42) was used to perform qRT‐PCR as per the manufacturer's protocol using 200 ng/μL RNA and gene‐specific TaqMan expression assays (ThermoFisher Scientific; cat. no #4351372) (Table S3). The relative RNA quantity was calculated using the delta–delta CT method, normalized to GAPDH as the standard control (6 biological repeats per treatment group).

### 2.6 Western Blotting

bEnd.3 cell lysates (*n* = 4 per group) were lysed in HEPES sample buffer containing 1% Triton‐X 100, 1% (v/v) protease inhibitor (Sigma; cat. no #P8340) and 1% (v/v) phosphatase inhibitor (Sigma; cat. no #P0044), sonicated for 10 s, and then centrifuged at 13000 rpm at 4°C for 10mins before supernatant collection. Protein concentrations of bEnd.3 lysates were measured with a Bradford protein assay (Bio‐Rad; cat. no #500‐0006) according to the manufacturer's protocol. Lysates (50 ng protein) were diluted in sample buffer and boiled for 5 min at 95°C, and western blotting was carried out as described previously (Baburamani et al. 2015). Protein lysates (50 ng) were separated by electrophoresis on a 4%–12% SDS‐PAGE gel (Invitrogen; cat. no #NP0326BOX) for 1 h at 180 V before transfer onto PVDF membrane (Millipore; cat. no #IPFL00010) for 2 h at 50V. Following transfer, membranes were blocked for 30 min in 10% milk diluted in Tris‐buffered saline containing 0.1% Tween‐20 (TBST). The membranes were cut according to molecular weight to allow for antibody probing of specific proteins. Primary antibodies were diluted in 10% milk and membranes were incubated in either STAT1 (1:1000, Cell Signalling; cat. no #14994S), pSTAT1 (1:500, Cell Signalling; cat. no #9167S), ICAM1 (1:1000, Invitrogen; cat. no #11844311), CXCL10 (1:500, R&D Systems; cat. no #AF‐466‐NA) or GAPDH (1:1000, Sigma Aldrich; cat. no #CB1001) primary antibody for 72 h at 4°C. Following TBST washes, membranes were incubated in the appropriate secondary antibody (Table S4) for 2 h in the dark at room temperature. Membranes were washed in TBST and then imaged and analysed using a LI‐COR imaging system and image studio (version 5.5.4). Fluorescence ratios (protein/GAPDH) were presented instead of normalising to control groups, as weak signal detection in PBS treated groups, due to low baseline protein levels of inflammatory mediators, limited reliable comparisons. This approach allows for more accurate quantification of protein expression across all samples, particularly when control signals are near or below detection limits.

### 2.7 Measurement of CXCL10

The concentration of CXCL10 secreted into the cell culture media was quantified at 4 and 24 h (*n* = 6) using the CXCL10 DuoSet ELISA development system (R&D Systems; cat. no #DY466) according to the manufacturer's instructions.

### 2.8 Caspase 3/7 Assay

Caspase 3/7 activity was analyzed 24 h post final treatment using the CellEvent Caspase‐3/7 green detection reagent (ThermoFisher Scientific; cat. no #C10723). bEnd.3 cells were incubated with reagent (5 μM) in complete medium for 30 min at 37°C and 5% CO_2_. Stained cells were then imaged and counted using the EVOS M5000 GFP filter cube, and counts normalized to total cell count acquired through brightfield images (×10 magnification). A total of 6 biological replicates with 3 technical replicates per group were performed.

### 2.9 Measurement of Cell Migration

Cells were seeded and treated in 12‐well plates as described above (*n* = 6, with 6 technical replicates). One hour after the final treatment was applied, a sterile 200 μL pipette tip was used to scratch down the centre of each well and fresh complete medium added. Cells were imaged immediately (0 h) using the EVOS M5000 cell imaging system (Fisher Scientific) at 10× magnification, and then reimaged 24 h later. Cell migration was determined by calculating the total area of the scratch wound in each image using the wound healing size tool plugin for ImageJ (Java 1.8.0_322) and data are presented as relative reduction in scratch area from average area at time 0.

### 2.10 Cell Proliferation Assay

Twenty‐four hours after the final treatment, bEnd.3 cells were incubated with 10 μg Hoechst (Sigma; cat. no #B2883) for 30 min. After incubation, the medium was removed, cells were washed briefly with PBS, and pre‐warmed fresh medium was added. The EVOS cell imaging system, with the DAPI imaging cube, was used to visualize fluorescent nuclei at 4× magnification (6 biological replicates, 3 technical replicates). Cells per image were counted using the automated cell counter on the EVOS M5000 software by defining the region of interest (nuclei) and the background.

### 2.11 Tube Formation Assay

To assess endothelial cell tubulogenesis, bEnd.3 cells were grown and treated in 6 cm cell culture dishes. One hour after the final treatment, cells were incubated for a further hour in serum‐deprived medium (DMEM + Pen/Strep + 1% FBS) and then trypsinised and plated into 96‐well plates (10 000 cells per well) coated with Gibco Geltrex LDEV‐Free Reduced Growth Factor Basement Membrane Matrix (Fisher Scientific; cat. no #A1413201). Vascular endothelial growth factor (VEGF; Fisher Scientific; cat. no #RB‐9031‐P0) was used as a positive control at a concentration of 25 ng/mL. Cells were incubated for 24 h, after which the centre of each well was imaged on the EVOS system (×40 magnification) and the number of branches in each image manually quantified and normalised to control (*n* = 3; mean of 6 technical replicates).

### 2.12 Statistical Analysis

The n values for all experiments were selected based on previous similar studies in bEnd.3 cells (Peng et al. 2011; Raoufi‐Rad et al. 2017; Charoensaensuk et al. 2021). The appropriateness of the selected sample sizes was confirmed using post hoc power calculations to confirm the achieved power (error probability 0.05) whereby all experiments had a power of > 0.9 aside from the first repeated inflammation cell death caspase 3/7 assay, which had a power of 0.73. Data were analysed using GraphPad PRISM (v.10.1.2). Outliers were tested for using the ROUT method (coefficient *Q* = 1%), and no outliers were identified. Normality was assessed using a Shapiro–Wilk test, which indicated no significant deviation from normality (*p* > 0.05) allowing us to proceed with parametric analysis. The statistical significance of mean differences between treatment groups was analysed using one‐way ANOVA followed by Tukey's post hoc analysis. A *p* value of < 0.05 was considered to be significant, and data are presented as mean ± standard deviation (SD).

## 3 Results

### 3.1 Bioinformatic Analysis Showed Consistent Alteration in TNF‐CXCL10 Pathway Activation in Cerebral Endothelial Cells Following Acute Inflammatory Stimulation

**FIGURE 1:**
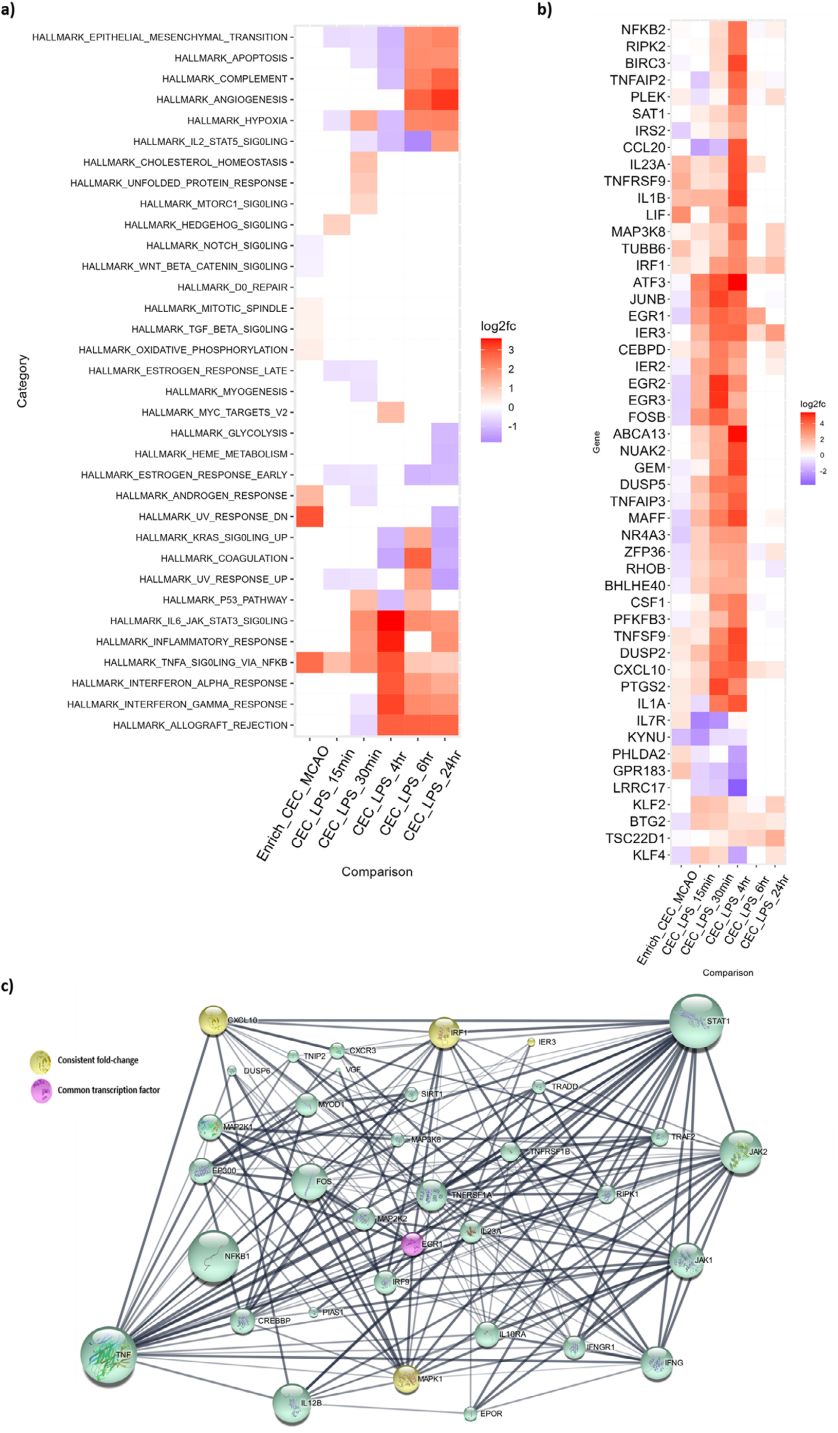
Different injurious stimuli assessed across multiple time points show consistent transcriptional alterations in the TNF via NFκB signaling pathway. Transcriptomic analysis was performed using published RNA‐Sequencing (RNA‐Seq) datasets. (a) Heatmap showing the average log‐2‐fold‐change (Log2FC) of gene sets significantly altered within the Hallmark Gene Set (*p*. adj < 0.05 for all comparisons). (b) Heatmap with hierarchical clustering of the genes with an over two‐fold change in expression in at least 4 comparisons within the Tumour necrosis factor (TNF) via Nuclear Factor‐κB (NFκB) signalling gene set. (c) STRING protein network analysis of 12 input genes, showing 20 first shell interactors and 5 s shell interactors. Line thickness corresponds to the degree of confidence prediction, and the size of each node equates to the number of protein–protein interactions within the network.

To investigate common alterations in cerebral endothelial cell gene expression between injury types and time frames, genomic enrichment analysis and differential gene expression were compared to their corresponding control. The log‐2‐fold‐change (Log2FC) of significantly altered gene sets (FDR < 0.05) was evaluated using EGSEA analysis. EGSEA analysis revealed consistent upregulation of the TNF via NF‐κB signalling category among all comparisons (Figure 1a).

To further investigate the specific genes within the TNF via NF‐κB gene set, the Log2FC of each significantly altered gene within the mouse genome was calculated and filtered to show the genes significantly upregulated (FDR < 0.05) in at least 4 comparisons. These data were plotted and displayed as a heatmap using ggdendro and ggplot (Figure 1b). Several genes were identified as being consistently altered in expression across comparisons. In particular, *Cxcl10*, Immediate Early Response 3 (*Ier3*) and Interferon Regulatory Factor 1 (*Irf1*) were shown to be consistently upregulated across all 6 comparisons. Multiple genes were upregulated in 5 of the 6 comparisons; see Table S5 for full list of identified genes in this category. The log2FC values show that the peak of most transcriptional changes occurs at the 4‐h time point. These candidates, along with genes that remaining upregulated at the latest analyzed time point of 24‐h, were among those identified for subsequent assessment. For this reason, both of these time points were selected for subsequent in vitro analysis.

Those genes identified as consistently altered across 4 comparison datasets were used as the input targets to generate a protein–protein interaction network using STRING (Figure 1c). TNF, STAT1, NF‐κB and Janus kinase 2 (JAK2) had the highest number of reported protein interactions within the network (29, 28, 26 and 22 interactions, respectively).

### 3.2 Repeated Low‐Level Inflammatory Challenge Alters Genes and Proteins Involved in TNF‐Induced Endothelial Activation

Our RNA‐Seq analysis supports the extensive body of literature showing that TNF stimulation leads to prolonged endothelial activation. Based on this, we next determined if repetitive low‐level TNF exposure altered the nature or extent of cerebral endothelial cell activation in comparison to a single acute treatment.

To select the concentration of TNF for use in a repeated challenge paradigm, a concentration‐response analysis was performed. bEnd.3 cells were treated with 0.05–10 ng/mL of TNF and the expression of *Icam1* was measured 4 h later as an indicator of endothelial cell activation. *Icam1* expression was significantly increased in the presence of TNF at 1, 5 and 10 ng/mL (*F*_(6,14) =_ 403.2, *p* = 0.0058, < 0.0001, < 0.0001, respectively) (Figure 1S). TNF at 0.5 ng/mL caused a 1.5 ± 0.08‐fold change, which did not reach statistical significance following post hoc testing (*F*_(6,14) =_ 403.2, *p* = 0.1140). This was therefore selected as a low ‘sub‐threshold’ TNF concentration for use in our repetitive injury model. TNF at 2.0 ng/mL was used as a positive control, as this is within the concentration range causing significant changes in *Icam1* expression and is the cumulative concentration of the four 0.5 ng/mL doses used for repeated challenge.

**FIGURE 2:**
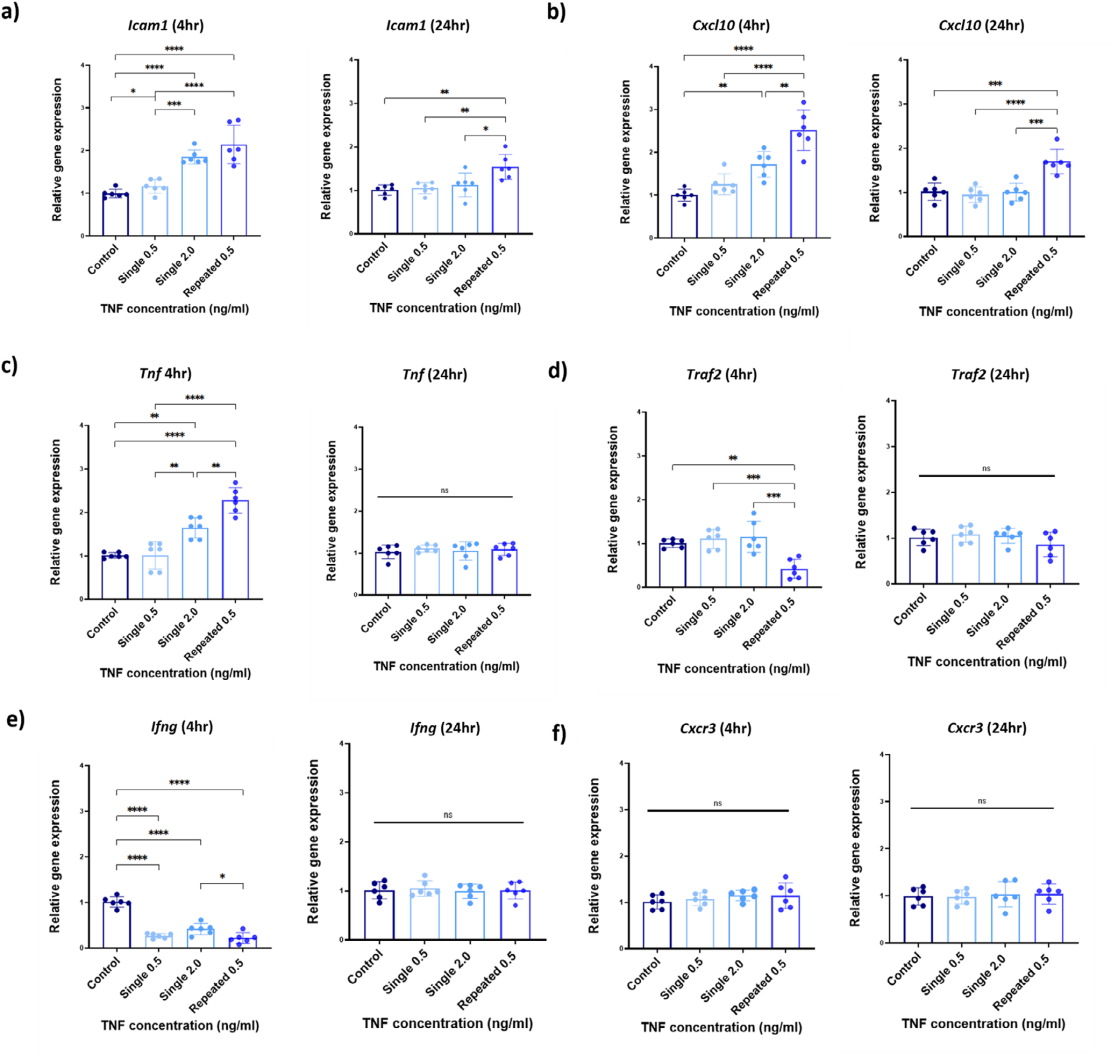
Repeated administration of a low concentration of TNF modifies the expression of genes associated with endothelial cell activation and the TNF‐CXCL10 pathway. qRT‐PCR was performed to quantify expression of the genes indicated in bEnd.3 cells in four treatment groups: Control (PBS), a single low concentration of Tumour necrosis factor (TNF) (0.5 ng/mL) for 1 h, a single cumulative concentration of TNF (2.0 ng/mL) for 1 h or repeated treatment with TNF at a low concentration (0.5 ng/mL) for 1 h on 4 consecutive days. Expression of Intercellular Adhesion Molecule 1 (*Icam1*) (a), C‐X‐C Motif Chemokine Ligand 10 *(Cxcl10)* (b), Tumour Necrosis Factor *(Tnf)* (c), TNF Receptor‐Associated Factor 2 *(Traf2)* (d), Interferon Gamma *(Ifng)* (e) and C‐X‐C Chemokine Receptor 3 *(Cxcr3)* (f) was quantified 4 h and 24 h post final treatment. Data were analysed using one‐way ANOVA, followed by Tukey's post hoc test. Biological replicates (*n* = 6) are expressed as mean ± SD. *p < 0.05, **p < 0.01, ***p < 0.001, ****p < 0.001. ns = not statistically significant.

bEND.3 cells were treated with 4 repeated low doses of TNF (0.5 ng/mL) over a 4‐day period. Gene and protein expression were then measured at 4 and 24 h. *Icam1* was shown to be significantly upregulated in response to repeated inflammation. A one‐way ANOVA showed a statistically significant effect of treatment group on *Icam1* expression at both 4 h post‐final treatment (*F*_(3,20) =_ 26.87, *p* < 0.0001) and 24 h post‐final treatment (*F*_(3,20) =_ 7.88, *p* = 0.0013). Post hoc analysis confirmed that *Icam1* expression was significantly higher in the repeated TNF treatment group compared to all other treatment groups (Figure 2a). Consistent with the concentration‐response data, there was no significant effect of a single 0.5 ng/mL dose of TNF on *Icam1* expression at 4 h compared to the PBS‐treated control (1.2 ± 0.2‐fold, *F*_(3,20) =_ 26.87, *p* = 0.6836), while the 2 ng/mL single TNF treatment increased *Icam1* expression by 1.9 ± 0.2‐fold (*F*_(3,20) =_ 26.87, *p* < 0.0001). By comparison, repeated low concentration TNF treatment significantly increased *Icam1* expression by 2.1 ± 0.5‐fold (*F*_(3,20) =_ 26.87, *p* < 0.0001 vs. PBS and 0.5 ng/mL groups) to a level similar to that caused by TNF at 2 ng/mL. After 24 h, *Icam1* expression in endothelial cells experiencing repeated TNF exposure was significantly higher than in all other treatment groups.

Of the TNF‐CXCL10 signalling pathway genes examined, *Cxcl10* and *Tnf* were both significantly increased at 4 h compared to all other treatment groups. This was shown to be 2.5 ± 0.5 fold for *Cxcl10* (*F*_(3,20) =_ 27.09, *p* < 0.0001) compared with PBS, and 2.3 ± 0.3 fold for *Tnf* (*F*_(3,20) =_ 35.95, *p* < 0.0001). Only *Cxcl10* remained significantly elevated at 24 h (*F*_(3,20) =_ 16.12, *p* = 0.0001) (Figure 2b,c). In contrast, expression of *Traf2* was not significantly modified by the 0.5 or 2 ng/mL single TNF treatments but was significantly downregulated (0.4 ± 0.2‐fold; *F*_(3,20) =_ 11.80, *p* = 0.0024) in the repeated challenge group compared to control at 4 h (Figure 2d). *Ifng* was significantly downregulated in all treatment groups compared to untreated control at 4 h (*F*_(3,20) =_ 69.75, *p* < 0.0001), returning to baseline at 24 h (*F*_(3,20) =_ 0.12, *p* = 0.9471; Figure 2e). There were no significant changes in expression of *Cxcr3*, CXCL10's primary receptor, across treatments at 4 h (*F*_(3,20) =_ 0.82, *p* = 0.4978) or 24 h (*F*_(3,20) =_ 0.1298, *p* = 0.9412; Figure 2f).

**FIGURE 3:**
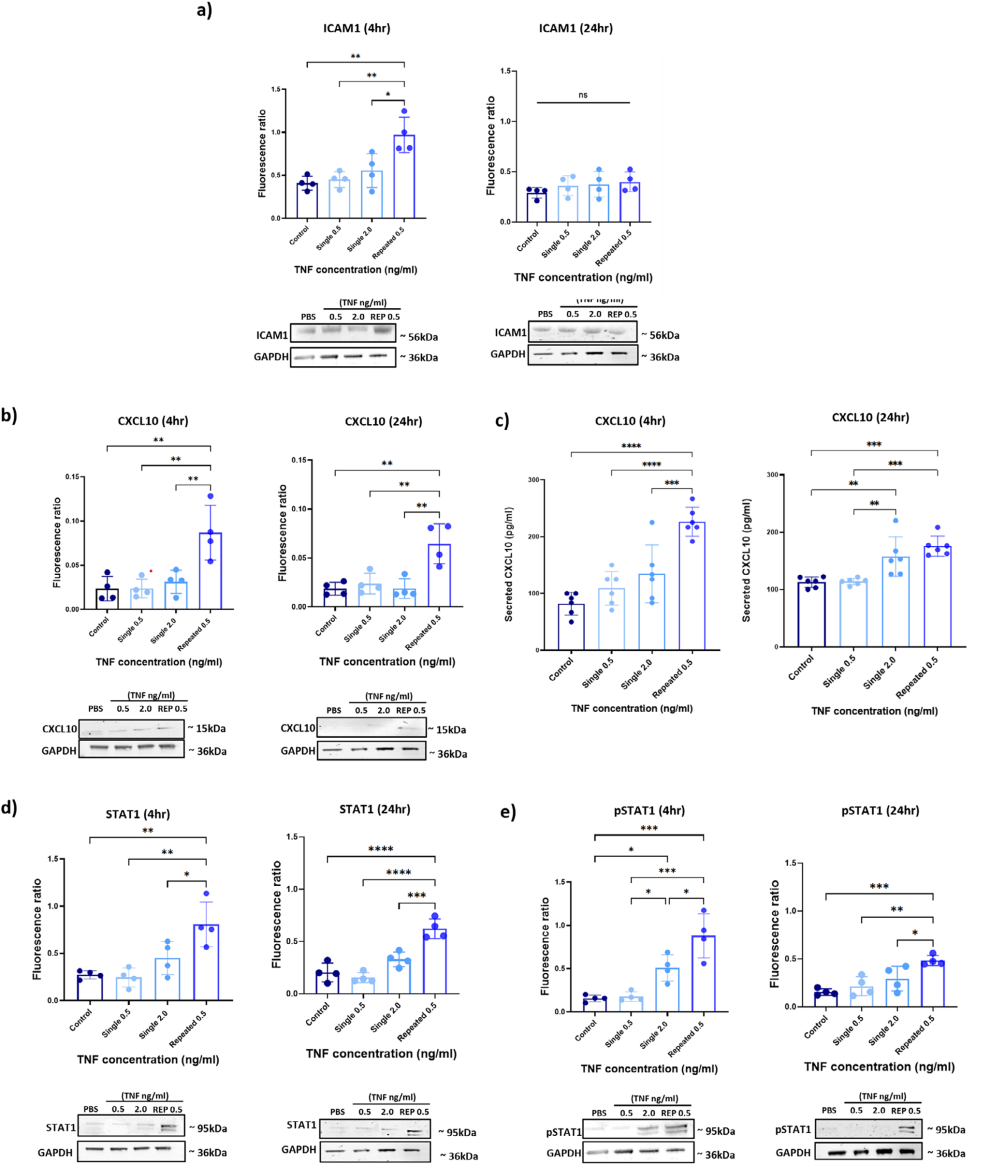
Repeated inflammatory challenge with TNF upregulates key proteins within the TNF‐CXCL10 pathway. bEnd.3 cells were exposed to control (phosphate buffered saline; PBS), a single low concentration of Tumour necrosis factor (TNF) (0.5 ng/mL), a single cumulative concentration of TNF (2.0 ng/mL) or repeated treatment with a low TNF concentration (0.5 ng/mL) for 1 h for 4 days. Intercellular Adhesion Molecule 1 (ICAM1) (a), C‐X‐C Motif Chemokine Ligand 10 (CXCL10) (b, c), Signal Transducer and Activator of Transcription 1 (STAT1) (d) and Phosphorylated STAT1 (pSTAT1) (e) expression were evaluated by western blotting at both 4 and 24 h post final treatment. Secreted CXCL10 protein was quantified using ELISA at both time points (c). Blots were run on the same gels and membranes, cut according to molecular weight and probed separately; therefore, the same GAPDH loading control is shown for some of these targets. Data were analysed using one‐way ANOVA, followed by Tukey's post hoc test. Biological replicates (*n* = 4) are given as mean ± SD. *p < 0.05, **p < 0.01, ***p < 0.001, ****p < 0.001.

Western blotting was used to determine if the observed changes in gene expression were mirrored at the protein level at both 4 and 24 h after repeated injury. ICAM1 was upregulated at 4 h compared to all other treatment groups (0.9 ± 0.2 for ICAM1 relative expression, *F*_(3,12) =_ 10.9, *p* = 0.0007 compared with 0.4 ± 0.08 in the PBS group) but was not significantly changed at 24 h (0.4 ± 0.1, *F*_(3,12) =_ 0.9, *p* = 0.46; Figure 3a). In keeping with the mRNA data, CXCL10 protein was increased in the repeated treatment group compared with all other groups at 4 (0.08 ± 0.03 for CXCL10 relative expression, *F*_(3,12)_ = 10.4, *p* = 0.0024 compared with 0.02 ± 0.01 in the PBS group) and 24 h post‐final treatment (0.06 ± 0.02 for CXCL10 relative expression, *F*_(3,12) =_ 11.7, *p* < 0.01 compared with all groups; Figure 3b). Secretion of CXCL10, as assessed by ELISA, was significantly increased at both 4 and 24 h following repeated TNF stimulation. Basal CXCL10 release was 90 ± 19 pg/mL and this was increased to 216 ± 25 (*F*_(3,20) =_ 20.88, *p* < 0.0001) and 153 ± 17 pg/mL (*F*_(3,20) =_ 14.81, *p* < 0.0001) at 4 and 24 h, respectively, in the repeated TNF treatment group (Figure 3c). A significant upregulation of STAT1 and pSTAT1 protein was found at both time points, with the relative phosphorylation of STAT1 peaking at 4 h in the repeated TNF treatment group (0.9 ± 0.3 compared to PBS control, *F*_(3,12) =_ 220.1, *p* = 0.0001, Figure 3d,e), significantly higher than with a single 2 ng/mL exposure. In summary, repeated exposure to a low, sub‐threshold concentration of TNF resulted in alteration of key genes and proteins involved in the TNF‐CXCL10 signalling axis, surpassing the effects of a single inflammatory challenge with a higher concentration of TNF.

### 3.3 Repeated Inflammatory Challenge Affects Cerebral Endothelial Cell Proliferation and Apoptosis

**FIGURE 4:**
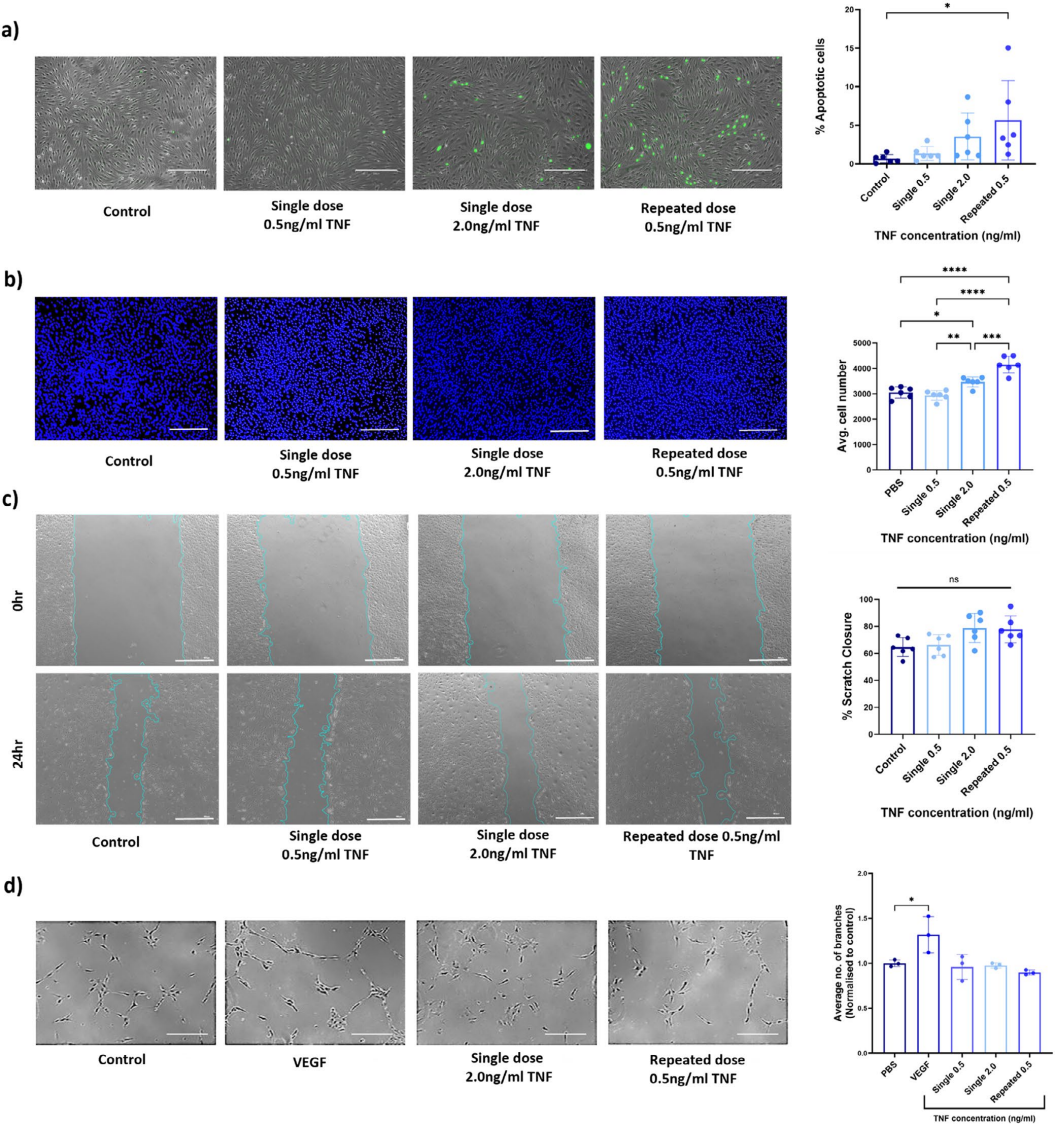
The effects of repeated low‐level inflammatory challenge on cerebral endothelial cell function. Functional analysis of bEnd.3 cells in response to repeated low‐level tumour necrosis factor (TNF) exposure compared to single low‐level and cumulative doses of TNF. (a) Caspase 3/7 activity was quantified 24 h post final TNF treatment; apoptotic (green) cells are shown against a bright field image (grey). The number of fluorescent cells per image was calculated as a percentage of total cell number (*n* = 6). Scale bar = 300 μm. (b) Cell proliferation was assessed by quantifying cell nuclei using Hoechst staining (blue) 24 h post final treatment (*n* = 6). Scale bar = 750 μm (c) bEnd.3 cell migration was assessed by scratch wound assay. Representative images at 0 and 24 h timepoints are shown. Combined data are shown as percentage scratch closure (*n* = 6). Scale bar = 300 μm. (d) The number of endothelial cell protrusions was counted per image for each condition and normalised to that of the control. Representative light microscope images of the tubular networks formed by bEnd.3 cells are shown. Vascular endothelial growth factor (VEGF) (25 ng/mL) was used as a positive control (*n* = 3). Scale bar = 75 μm. Data were analysed using one‐way ANOVA, followed by Tukey's post hoc test. Biological replicates are presented as mean ± SD. *p < 0.05, **p < 0.01 and ***p < 0.001, ****p < 0.0001. ns = not statistically significant.

To investigate whether the altered inflammatory signalling seen in the repeated TNF treatment group was associated with changed endothelial cell function, we assessed bEnd.3 cell apoptosis, proliferation, migration and ‘tube’ formation 24 h after treatment. Measurement of cleaved caspase3/7 activity identified a significant increase in caspase 3/7 activity in the repeated TNF challenge group (5.64% ± 5.14%) 24 h post‐final treatment when compared to control (1.35% ± 0.54%; *F*_(3,20) =_ 3.317, *p* = 0.0196), but there was no difference between the repeated exposure group and the single exposure 2.0 ng/mL TNF group (3.54 ± 3.03, *p* = 0.6015; Figure 4a). Endothelial cell proliferation, as assessed by nuclear Hoechst staining, showed a significant increase in cell number in repeatedly treated cells (4149 ± 327 cells) 24 h post‐final treatment compared to PBS controls (3045 ± 221 cells, *F*_(3,20) =_ 31.15, *p* < 0.0001), the single 0.5 ng/mL group (2937 ± 191, *F*_(3,20) =_ 31.15, *p* < 0.0001) and single 2.0 ng/mL treated cells (3471 ± 196 cells, *F*_(3,20) =_ 31.15, *p* = 0.0005) (Figure 4b). No significant difference was found between single TNF challenges or repeated exposure to TNF on the rate of scratch wound closure within the time frame of these experiments, indicating no effect on directional migration (*F*_(3,20) =_ 4.176, *p* = 0.0695; Figure 4c). Similarly, analysis of bEnd.3 cell tubulogenesis showed no effect of any TNF treatment group, whereas VEGF (positive control) significantly enhanced tube formation compared to the PBS control (*F*_(3,20) =_ 6.502, *p* = 0.0194; Figure 4d). Thus, repeated low‐level inflammatory challenge in vitro increases bEnd.3 cell apoptosis and proliferation but has no impact on endothelial cell migration or angiogenic potential.

### 3.4 Knockdown of CXCL10 in Cerebral Endothelial Cells Reduced the caspase3/7 Activity Induced by Repeated Inflammatory Challenge

As CXCL10 was upregulated at the gene and protein levels at both 4 and 24 h in bEnd.3 cells following repeated TNF exposure, we hypothesised that the elevated CXCL10 may be contributing to the functional outcomes observed in brain endothelial cells. First, to validate the increased CXCL10 expression observed in bEnd.3 cells following repeated inflammation, we conducted a preliminary analysis using the same TNF concentrations and repetitive exposure model in a human brain endothelial cell line (HBEC‐5i). Consistent with the response shown in bEnd.3 cells, there was an exaggerated increase in CXCL10 gene expression in HBEC‐5i cells in response to repeated inflammatory stimulation compared to all control groups (Figure S3).

**FIGURE 5:**
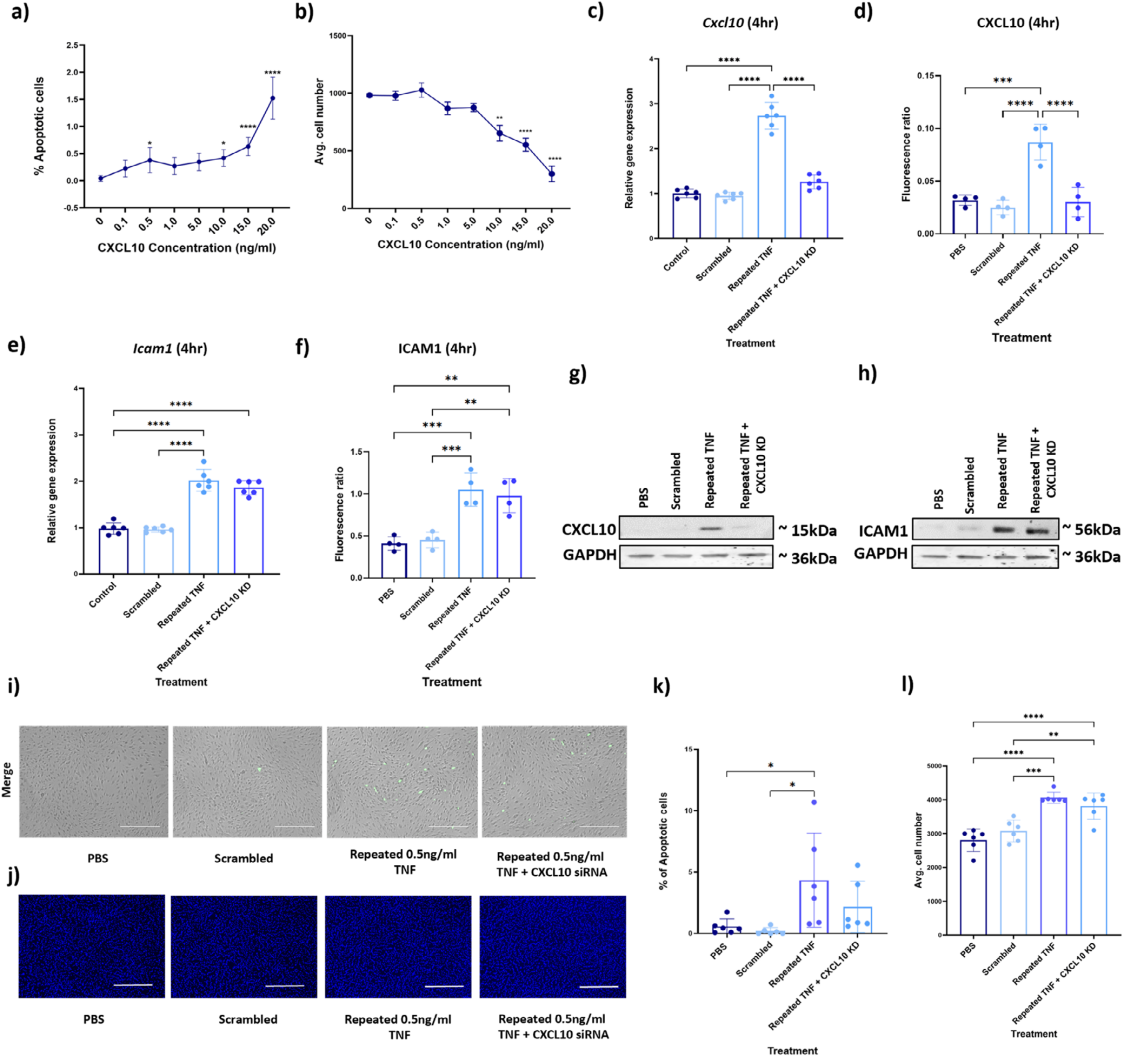
CXCL10 influences apoptosis and proliferation of bEnd.3 cells. (a) bEnd.3 cells were treated for 24 h with the indicated C‐X‐C Motif Chemokine Ligand 10 (CXCL10) concentrations and caspase 3/7 activity measured, with percentage apoptosis calculated from the number of fluorescent cells relative to total cell number. (b) Cell nuclei were stained with Hoechst following CXCL10 treatment, and cell number was quantified and normalised to control. (c–h) Cells treated with CXCL10‐targeting small interfering RNA (siRNA) or scrambled control siRNA were repeatedly exposed to Tumour necrosis factor (TNF) (0.5 ng/mL). Gene and protein expression of CXCL10 and ICAM1 was measured using RT‐qPCR and western blotting. (i) Representative light and fluorescent images (10× magnification) of cells treated with either CXCL10‐targeting siRNA or scrambled control siRNA and subsequently exposed to TNF (0.5 ng/mL), showing apoptotic (green) cells. Scale bar = 300 μm. (j) Representative images of Hoechst staining (blue) for quantification of cell number in the same treated cells. Scale bar = 750 μm. (k) The number of apoptotic cells per image was calculated as a percentage of total cell number. (l) The mean cell number was calculated for each treatment group. Data were analysed using one‐way ANOVA, followed by Tukey's post hoc test. Biological replicates (*n* = 4–6) are shown as mean ± SD. *p < 0.05, **p < 0.01 and ***p < 0.001 and ****p < 0.0001.

To test the hypothesised functional contribution of CXCL10, bEnd.3 cells were treated with recombinant CXCL10 protein at a range of concentrations (0.1–20 ng/mL), and apoptosis and proliferation were quantified after 24 h (Figure 5a,b). A concentration‐related increase in the number of apoptotic cells was found following exposure to CXCL10, with the highest percentage of apoptotic cells (1.5%) seen at 20 ng/mL (*F*_(7,16) =_ 29.74, *p* < 0.0001; Figure 5a). Treatment with CXCL10 also significantly decreased cell number at the higher concentrations of 10, 15 and 20 ng/mL (*F*_(7,16) =_ 70.55, *p* < 0.0001; Figure 5b).

To determine if elevated CXCL10 contributed to the cellular changes following repetitive TNF exposure, CXCL10 was knocked down in bEnd.3 cells using siRNA (Figure S4). Cells were transfected with an siRNA targeting CXCL10 or a non‐targeting control (scrambled siRNA), and then repeatedly exposed to TNF (0.5 ng/mL) or PBS, as described earlier. Consistent with successful CXCL10 knockdown, no elevation in *Cxcl10* mRNA (*F*_(3,20) =_ 96.79, *p* = 0.1798) or protein (*F*_(3,12) =_ 20.3, *p* = 0.25) was observed following repeated TNF treatment compared to control at the 4 h timepoint (Figure 5c,d,g). Silencing of CXCL10, however, had no effect on the increased *Icam1* mRNA (*F*_(3,20) =_ 58.93, *p* < 0.0001) and protein (*F*_(3,12) =_ 14.76, *p* = 0.0036) expression induced by repeated exposure to TNF after 4 h (Figure 5e,f,h), suggesting no role for CXCL10 in stimulating ICAM1 upregulation. In keeping with our previous data (Figure 4a), the repeated TNF stimulation group showed an increased percentage of apoptotic cells compared to all controls (*F*_(3,20) =_ 4.35, *p* = 0.0369). The mean percentage caspase 3/7 activity was less when repeated TNF stimulation was combined with CXCL10 knockdown, with no significant difference between this group and the PBS‐ or scrambled siRNA‐treated controls (*F*_(3,20) =_ 4.35, *p* = 0.6 and 0.4, respectively). The significant increase in proliferation observed in the presence of repeated TNF stimulation (see also Figure 4b) was unaffected by CXCL10 knockdown (*F*_(3,20) =_ 11.65, *p* = 0.9993; Figure 5j,l). These data, combined with the observed decreases in proliferation following exogenous CXCL10 administration, suggest that CXCL10 does not contribute to the increased proliferation observed with repetitive low‐level TNF treatment.

## 4 Discussion

In this study we investigated the responses of brain endothelial cells in vitro to repeated inflammatory challenge. The results of our bioinformatic analysis revealed the continuous upregulation of the TNF (via NF‐κB) signaling pathway in the cerebral endothelium in response to different injury conditions and timeframes. Our in vitro findings demonstrate, for the first time, that repeated low‐level inflammatory insult, driven by TNF, triggers an alteration in the cerebral endothelial cell response that could contribute to injury in the brain vasculature.

In line with the literature from peripheral endothelial cells (e.g., Kempe et al. 2005; Pan et al. 2016; Zhou et al. 2017), our transcriptomic analysis identified a consistent upregulation of the TNF (via NF‐κB) signaling pathway in the cerebral endothelial cells from an acute model of stroke and LPS stimulation from 15min to 24 h. Exploration of the gene‐specific changes occurring in these transcriptomic comparisons highlighted an increase in TNF‐CXCL10 signaling. In the brain specifically, these findings are supported by a recent transcriptomic study showing upregulation of TNF expression and NF‐κB signaling in response to cerebral endothelial cell injury in vitro and in an in vivo middle cerebral artery occlusion (MCAO) model of stroke, with TNF upregulation persisting for 24 h post‐MCAO injury (Ji et al. 2024). Interestingly, distinct phases of altered gene expression profiles (1–4 h and 12–24 h) have been reported in transcriptomic studies of human umbilical vein endothelial cells (HUVECs) in response to TNF exposure, with specific genes (*Irf1* and *Cxcl10*) more highly upregulated with the longer TNF exposure (Struck et al. 2024). Whether such time‐specific responses occur in cerebral endothelial cells remains an unanswered question.

We hypothesised that the consistent upregulation of the primary mediators of the TNF‐CXCL10 signalling pathway, in response to inflammatory challenge, was indicative of their fundamental importance in regulating endothelial cell responses. Moreover, excessive or prolonged activation of these signalling pathways may drive endothelial cell dysfunction (Steyers and Miller 2014). In support of this, we observed a sustained upregulation of CXCL10, STAT1 and pSTAT1 at 4 and 24 h after repeated exposure to a low TNF concentration, compared to controls. CXCL10 is a small chemoattractant, typically acting via its primary receptor CXCR3 in response to pro‐inflammatory conditions (van den Borne et al. 2014). CXCR3 is expressed on immune cells, including monocytes and T cells. Its activation by CXCL10 leads to activation of downstream signalling pathways such as PI3K‐Akt, MAPK and NF‐κB, driving cellular migration, adhesion, and inflammatory responses (discussed in detail by Liu et al. 2011). This interaction between CXCL10 and CXCR3 plays a crucial role in immune recruitment and chemoattraction (Tokunaga et al. 2018).

Investigation into the contribution of CXCL10 in neurological disease has identified elevated levels of CXCL10 to be present in multiple sclerosis, Alzheimer's disease, and cerebral malaria (Xia et al. 2000; Sørensen et al. 2002; Wilson et al. 2011; Zaheer et al. 2013). Moreover, studies in animal models of multiple sclerosis have shown the TNF‐STAT1‐CXCL10 axis to contribute to pathology, and it has been suggested as a promising therapeutic target (Rahmat‐Zaie et al. 2023).

Increases in cerebrovascular expression of CXCL10 have been shown to be primarily mediated by IFNγ (Wang et al. 2016; Sorensen et al. 2018). While TNF is known to activate CXCL10 gene transcription, typically via the NF‐κB pathway, this has not, until now, been explored in cerebral endothelial cells. In non‐brain endothelial cells, TNF‐induced expression of interferon‐stimulated genes, such as CXCL10, has been reported to be dependent on IRF1 production and consequent autocrine STAT1 signalling (Venkatesh et al. 2013). As STAT1 is an established upstream regulator of CXCL10 (Sikorski et al. 2011), our findings that both CXCL10 and STAT1 remain upregulated for sustained periods, and that IFNγ is downregulated with repeated TNF exposure, support the idea that this pathway is activated with repetitive low‐level inflammatory stimulation.

In addition to enhanced levels of CXCL10, STAT1 and ICAM1 with repeated TNF exposure, we also found a significant decrease in *Traf2* expression in response to repeated TNF at 4 h post final treatment. TRAF2 is a ubiquitously expressed scaffold protein, established as a regulator of inflammation, usually activated directly by TNF and linked to the activation of NF‐κB and other downstream pathways (Xie 2013). Downregulation of TRAF2 concomitant with increased expression of CXCL10 and STAT1 could be explained by an immune tolerance mechanism, induced to facilitate the resolution of inflammation (Lalani et al. 2018).

In addition to the known functions of CXCL10 and ICAM1 in leukocyte recruitment to and across the endothelium, pro‐inflammatory cytokines and downstream NF‐κB signalling pathways have been linked to alterations in proliferation (Mehta et al. 2018; Khan et al. 2023) and apoptosis (Rastogi et al. 2012; Chen et al. 2019; Mussbacher et al. 2019; Otano et al. 2020; van Loo and Bertrand 2023). In this study, repetitive low‐level inflammatory challenge of brain endothelial cells with TNF resulted in increased proliferation and increased apoptosis, findings that have not, to our knowledge, been reported previously. Previous studies focussed on non‐brain endothelial cells and showed that TNF decreases endothelial cell proliferation in vitro (Rastogi et al. 2012; Barros Ferreira et al. 2024). It has been suggested that this likely results from its well‐established pro‐apoptotic effects, as opposed to a direct proliferative effect (e.g., Sawant et al. 2013). However, treatment of human brain endothelial cells with TNF (up to 10 ng/mL) showed an upregulation in proliferation that was ameliorated in the presence of TNFR1 neutralising antibodies (Huang et al. 2016). The single cumulative concentration of TNF used here (2 ng/mL) had a small effect on proliferation and did not significantly affect apoptosis, suggesting that the upregulation of down‐stream signalling pathways occurring after repeated TNF exposure was required to induce alterations in these cellular responses. With respect to the actions of CXCL10, published data suggest that this chemokine has anti‐proliferative effects on microvascular endothelial cells, independently of CXCR3 engagement (Luster et al. 1995; Campanella et al. 2010), although there are no existing reports of a similar effect in brain endothelial cells. Our data in CXCL10‐stimulated bEnd.3 cells support the notion that CXCL10 exerts anti‐proliferative actions on brain endothelial cells. The concentrations of CXCL10 secreted from bEnd.3 cells following repeated TNF exposure were 10‐fold lower than required to reduce proliferation with exogenous recombinant CXCL10, suggesting that autocrine control of proliferation following TNF exposure in vitro is unlikely. If CXCL10 contributes to reduced proliferation, it may be through an altered, combined action with the other mediators produced following repeated TNF stimulation. However, our results also showed that siRNA‐mediated knockdown of CXCL10 had no effect on TNF‐stimulated proliferation, supporting the idea of a CXCL10‐independent control of proliferation in cells experiencing repeated TNF exposure. Further investigation of the TNF‐STAT1‐CXCL10 signalling pathway is warranted to identify the regulators of endothelial proliferation in this model.

Here, increased caspase 3/7 activity was observed in response to repeated inflammatory challenge when compared with PBS and cells treated with a single TNF dose (0.5 ng/mL), though not statistically different from that of a single 2.0 ng/mL TNF. In the absence of CXCL10, there was a trend toward lower caspase 3/7 activity in response to repeated inflammation, though this was not found to be statistically significant. While it is possible from these data that CXCL10 does not contribute to the apoptosis seen in the repeated exposure group, the lack of significance may reflect the high variability in the caspase 3/7 response and suggests that further studies with larger sample sizes (e.g., *n* = 12 to achieve 0.9 power) are warranted to better evaluate this effect. CXCL10 can promote apoptosis in various cell types, including T lymphocytes (Sidahmed et al. 2012), neurons (Sui et al. 2004) and HUVECs (Feldman et al. 2006). Here, we showed that treatment of bEnd.3 cells with recombinant CXCL10 for 24 h increased apoptosis in a concentration‐dependent manner, with a significant effect observed at a low concentration (500 pg/mL). However, the degree of apoptosis observed with CXCL10 at 500 pg/mL was less than that seen in the repetitive TNF exposure model, even though the concentration of CXCL10 secreted was lower (~150 pg/mL over a 24‐h period). These findings suggest that CXCL10 can contribute to apoptosis, but that other, unidentified, pro‐apoptotic mediators may be working alongside it to increase endothelial cell apoptosis in response to repeated inflammatory challenge. In this respect, our transcriptomic data analysis showed a relatively consistent upregulation (4/6 comparisons) of the common transcription factor *EGR1* in cerebral endothelial cells in response to injury. This may be one possible candidate, as EGR1 has been shown to promote endothelial cell apoptosis, albeit through undefined mechanisms (Xie et al. 2023).

Previous in vitro investigation has shown increased tube formation by bEnd.3 cells in response to a single TNF treatment of 100 ng/mL (Yuen et al. 2014), 50‐fold greater than the highest concentration used in the present study. Similarly, primary rat cerebral endothelial cells show increased tube formation in response to TNF acting via TNFR1‐NF‐κB signalling (Wang et al. 2011). In the latter study, significantly increased tubulogenesis was shown in response to 10 ng/mL, but not 2.5 ng/mL or 5 ng/mL TNF (Wang et al. 2011), similar to the lack of effect observed in our in vitro model employing low TNF concentrations. in vivo studies also suggest a pro‐angiogenic effect of TNF on the cerebral microvasculature, occurring through TNFR1‐mediated pathways (Huang et al. 2016). No significant changes in endothelial ‘tube’ formation or cell migration were found in response to repeated challenge with TNF, suggesting that these bEnd.3 cell functions are not impacted by this treatment regimen.

A limitation of this study is the use of an immortalised mouse cerebral endothelial cell line. This cell population was chosen to align with the RNA‐Seq analysis but does limit the translatability of the study to the human blood–brain barrier. However, these findings provide a valuable proof‐of‐concept for understanding the endothelial‐specific response, with preliminary data from a human endothelial cell line further supporting these results. Future studies incorporating co‐culture models, primary human cells, or in vivo approaches would be valuable for translating these findings to the entire blood–brain barrier.

This study is the first to investigate the response of cerebral endothelial cells to repeated low‐level inflammatory challenge. These results demonstrate increases in CXCL10 and relative STAT1 phosphorylation following repeated exposure to TNF, alongside increased apoptosis and proliferation, persisting for 24 h after the final repeated challenge. This suggests a longer lasting regulatory influence of inflammation that may ultimately contribute to prolonged dysfunction of the cerebral endothelium. Future studies are warranted to fully understand how the TNF‐STAT1‐CXCL10 signaling axis influences the response of cerebral endothelial cells to recurrent inflammation.

## Author Contributions

**Megan Ritson:** conceptualization, methodology, investigation, formal analysis, writing – original draft, writing – review and editing. **Dong Xia:** conceptualization, methodology, supervision, writing – review and editing. **Caroline Wheeler‐Jones:** conceptualization, methodology, supervision, resources, writing – review and editing, funding acquisition. **Helen B. Stolp:** conceptualization, methodology, supervision, funding acquisition, resources, project administration, writing – review and editing, writing – original draft.

## Ethics Statement

The authors have nothing to report.

## Consent

The authors have nothing to report.

## Conflicts of Interest

The authors declare no conflicts of interest.

## Peer Review

The peer review history for this article is available at https://www.webofscience.com/api/gateway/wos/peer‐review/10.1111/jnc.70130.

## Supporting information


Data S1.

Data S2.

## Data Availability

All raw data is available upon request. All data generated or analysed during this study are included in this article. Further enquiries can be directed to the corresponding author.
