# Supplementary material for: Repeated Low‐Level Inflammatory Challenge Leads to Alterations in the TNF‐CXCL10 Signalling Pathway in Mouse Cerebral Endothelial Cells In Vitro
Source: J Neurochem. 2025 Jun 16;169(6):e70130. doi: 10.1111/jnc.70130 (PMC12169089; doi:10.1111/jnc.70130)
Supplement: Supplementary file 1 — Data S1. [file JNC-169-0-s002.pdf]

Repeated low-level inflammatory challenge leads to alterations in the TNF-CXCL10 signalling pathway in mouse cerebral endothelial cells *in vitro.*

Megan Ritson[1], Dong Xia[1], Caroline Wheeler-Jones[1] and Helen B. Stolp[1]*

[1]Department of Comparative Biomedical Sciences, Royal Veterinary College, London NW1 0TU

*__Corresponding Author__:

H.B. Stolp

Department of Comparative Biomedical Sciences

Royal Veterinary College

Royal College Street, London NW1 0TU, UK

hstolp@rvc.ac.uk

ORCID ID: 0000-0001-6019-5426

Author email addresses: mritson7@rvc.ac.uk, dxia@rvc.ac.uk, cwheeler@rvc.ac.uk, hstolp@rvc.ac.uk

Repeated low-level inflammatory challenge leads to alterations in the TNF-CXCL10 signalling pathway in mouse cerebral endothelial cells *in vitro*

Megan Ritson[1], Dong Xia[1], Caroline Wheeler-Jones[1] and Helen B. Stolp[1]*

Supplementary methods

Cell culture of HBEC-5i cell line

The human brain endothelial cell line, HBEC-5i, was purchased from ATCC. This cell line derives from the cerebral cortex of a patient. The cells were thawed in a 37°C water bath and seeded into a T75 culture flask pre-coated with 0.1% gelatin (ATCC® PCS-999-027). The flask contained complete medium composed of DMEM:F12 (ATCC® 30-2006™) supplemented 107 with 2.5 mM L-glutamine, 15 mM HEPES, 0.5 mM sodium pyruvate, and 1200 mg/L sodium bicarbonate. Additionally, the medium was supplemented with 40 µg/mL endothelial cell growth supplement (ECGS), 10% fetal bovine serum (FBS; ATCC® 30-2020), and 2% penicillin/streptomycin (Gibco; #15140122). Cells were grown to ~90% confluency, trypsinised (Trypsin/0.25% EDTA; Thermo Scientific; #25200056), and then seeded at a ratio of 1:4. Cells were used between passages 2 and 6 and plated at a density of 8 x 10^4 (12-well plate) to meet the required level of confluency.

Treatments for repeated injury experiments and RNA extraction protocols were the same as that used in the bEnd.3 cells. For qRT-PCR, the *Cxcl10* (Hs00171042_m1) and *Gapdh* (Hs02786624_g1) TaqMan primers were used to quantify gene expression.

Repeated low-level inflammatory challenge leads to alterations in the TNF-CXCL10 signalling pathway in mouse cerebral endothelial cells *in vitro*

Megan Ritson[1], Dong Xia[1], Caroline Wheeler-Jones[1] and Helen B. Stolp[1]*

Supplementary Data

Supplementary Table 1: RNA-Seq datasets used for analysis

Study	Species	Cell type	Injury stimulus	Comparisons of interest	Control
Munji et al, 2019	*Mouse*	Enriched brain endothelial cells<br><br>Derived from VE-Cadherin-Cre$_{ERT2}$ mice.	Middle Cerebral Artery Occlusion (MCAO) – Acute stroke model	Acute stroke vs control, tissues harvested at 24hrs.	Enriched brain endothelial cells from untreated mice
Kodali et al, 2020	*Mouse*	Cerebral endothelial cells<br><br>Derived from 8–12-week-old male and female C57BL/6 mice.	Lipopolysaccharide *(Escherichia coli* O55:B5)<br><br>10mg/kg	LPS 15min vs control<br><br>LPS 30min vs control<br><br>LPS 4hr vs control	Cerebral endothelial cells from PBS injected mice
Jambusaria et al, 2020	*Mouse*	Cerebral endothelial cells<br><br>Derived from RiboTag^{EC} (*Cdh5* $^{CreERT2/+}$; *Rpl22*$^{HA/+}$) mice.	Lipopolysaccharide *(Escherichia coli* O55:B5)<br><br>10mg/kg	LPS 6hr vs control<br><br>LPS 24hr vs control	Cerebral endothelial cells from PBS injected mice

Supplementary Table 2: Mouse CXCL10 siRNAs

siRNA Mouse CXCL10	Target sequence	Nanomoles	Micrograms
**siRNA 1 D-042605-01**	CCCAAGUGCUGCCGUCAUU	2.0	26.8
**siRNA 2 D-042605-03**	AGAGAUGUCUGAAUCCGGA	2.0	26.8
**siRNA 3 D-042605-03**	CCAUAGGGAAGCUUGAAAU	2.0	26.7
**siRNA 4 D-042605-04**	CCAUAUCGAUGACGGGCCA	2.0	26.8

Repeated low-level inflammatory challenge leads to alterations in the TNF-CXCL10 signalling pathway in mouse cerebral endothelial cells *in vitro*

Megan Ritson[1], Dong Xia[1], Caroline Wheeler-Jones[1] and Helen B. Stolp[1]*

Supplementary Table 3: TaqMan primers used for qPCR analysis

Gene name	TaqMan accession	Probe location (exon boundary)
GAPDH	Mm99999915_g1	2-3
TNF	Mm00443258_m1	1-2
CXCL10	Mm00445235_m1	1-2
IFNG	Mm01168134_m1	3-4
TRAF2	Mm00801978_m1	4-5
CXCR3	Mm99999054_s1	2
ICAM1	Mm00516023_m1	2-3

Supplementary Table 4. Secondary antibodies used for western blotting

IRDye 800CW	Supplier	Concentration
**Goat anti-mouse**	Li-Cor; 926-32210	1:10,000
**Goat anti-rabbit**	Li-Cor; 926-32211	1:10,000
**Donkey anti-goat**	Li-Cor; 926-32214	1:10,000

Repeated low-level inflammatory challenge leads to alterations in the TNF-CXCL10 signalling pathway in mouse cerebral endothelial cells *in vitro*

Megan Ritson[1], Dong Xia[1], Caroline Wheeler-Jones[1] and Helen B. Stolp[1]*

Supplementary Table 5: Genes showing transcriptomic alterations in 5 or more comparisons

Gene name	Gene symbol
**C-X-C motif chemokine ligand 10**	*CXCL10*
**Early Growth Response 1**	*EGR1*
**Ras Homolog Family Member B**	*RHOB*
**BTG Anti-Proliferation Factor 2**	*BTG2*
**ZFP36 Ring Finger Protein**	*ZFP36*
**KLF Transcription Factor 4**	*KLF4*
**MAF BZIP Transcription Factor F**	*MAFF*
**Immediate Early Response 2**	*IER2*
**KLF Transcription Factor 2**	*KLF2*
**6-Phosphofructo-2-Kinase/Fructose-2,6-Biphosphatase 3**	*PFKFB3*
**TSC22 Domain Family Member 1**	*TSC22D1*
**Interferon Regulatory Factor 1**	*IRF1*
**TNF Alpha Induced Protein 2**	*TNFAIP2*
**CCAAT Enhancer Binding Protein Delta**	*CEBPD*
**Pleckstrin**	*PLEK*
**Interleukin 23 Subunit Alpha**	*IL23A*
**Colony Stimulating Factor 1**	*CSF1*
**Immediate Early Response 3**	*IER3*
**Nuclear Factor Kappa B Subunit 2**	*NFKB2*
**JunB Proto-Oncogene, AP-1 Transcription Factor Subunit**	*JUNB*
**Mitogen-Activated Protein Kinase Kinase Kinase 8**	*MAP3K8*
**Tubulin Beta 6 Class V**	*TUBB6*

Repeated low-level inflammatory challenge leads to alterations in the TNF-CXCL10 signalling pathway in mouse cerebral endothelial cells *in vitro*

Megan Ritson[1], Dong Xia[1], Caroline Wheeler-Jones[1] and Helen B. Stolp[1]*

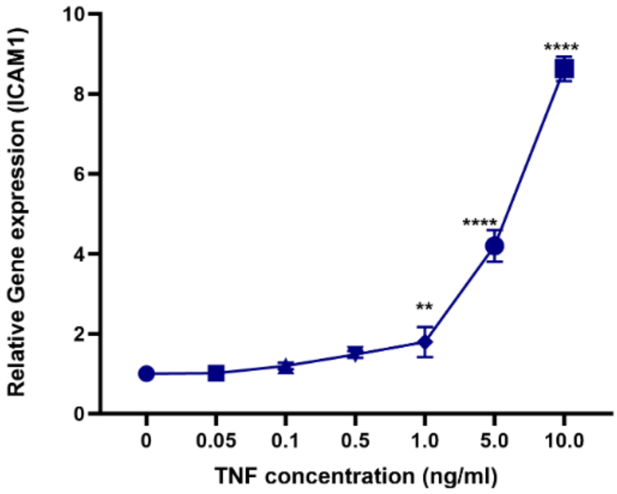

Supplementary Figure 1. TNF induces concentration-dependent changes in *ICAM1* mRNA expression.

bEnd.3 cells were treated with a range of Tumor necrosis factor (TNF) concentrations for 1 hour and expression of Intercellular Adhesion Molecule 1 (*Icam1)* was quantified via qRT-PCR after 4 hours. A significant increase in *Icam1* gene expression was found with TNF concentrations above 1ng/ml, but not when cells were treated with 0.5ng/ml TNF or lower. Data were analysed using one-way ANOVA, followed by Tukey's post hoc test. Biological replicates (n=3) are shown as mean ± SD. ** $P < 0.01$, **** $P < 0.001$.

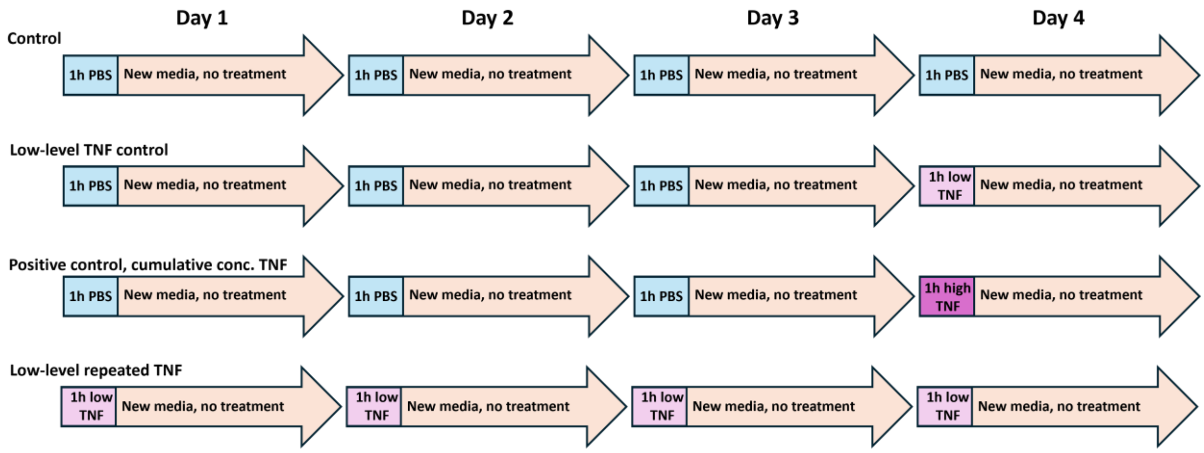

Supplementary Figure 2. Schematic of the repeated injury treatment method design.

bEnd.3 cells were seeded on the appropriate culture plates for 24 hours before being treated on day 1. For the repeated injury experimental group, cells were treated with 0.5ng/mL Tumor necrosis factor (TNF) every day for 1 hour per day with fresh medium added after removal of the stimulus. The other groups were treated as indicated with an equivalent volume of phosphate buffered saline (PBS), or with either a low dose of TNF (0.5ng/mL) or a cumulative dose of TNF (2.0ng/mL) on day 4 Protein extraction, RNA gene extraction and functional analyses were carried out either 4hours or 24hours post-final treatment.

Repeated low-level inflammatory challenge leads to alterations in the TNF-CXCL10 signalling pathway in mouse cerebral endothelial cells *in vitro*

Megan Ritson[1], Dong Xia[1], Caroline Wheeler-Jones[1] and Helen B. Stolp[1]*

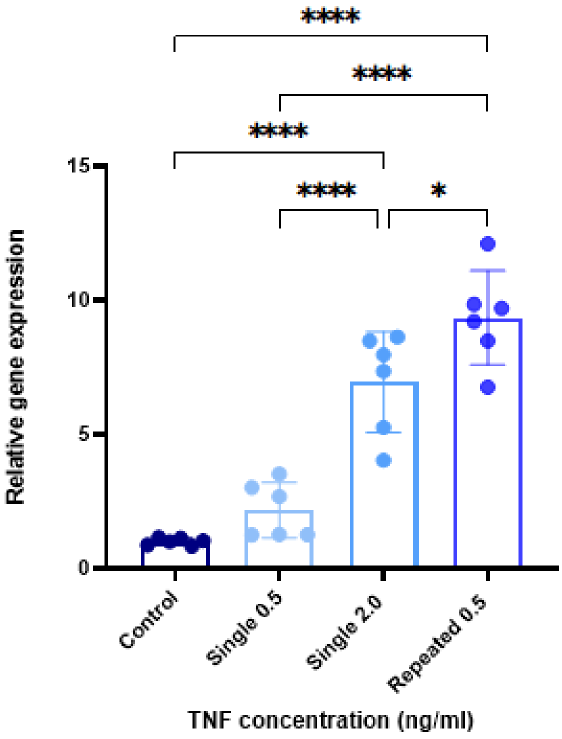

Supplementary Figure 3. Response of human cerebral endothelial cell line to repeated low-level inflammation.

qRT-PCR was performed to quantify expression of the C-X-C Motif Chemokine Ligand 10 *(Cxcl10)* in HBEC-5i cells in four treatment groups: control (PBS), a single low concentration of Tumor necrosis factor (TNF) (0.5ng/mL) for 1 hour, a single cumulative concentration of TNF (2.0ng/mL) for 1 hour or repeated treatment with TNF at a low concentration (0.5ng/mL) for 1 hour on 4 consecutive days. Expression of *Cxcl10* was quantified 4 hours post final treatment. Data were analysed using one-way ANOVA, followed by Tukey's post hoc test. Biological replicates (n=6) are expressed as mean ± SD. *p<0.05, **p<0.01, ***p<0.001, ****p<0.001. ns = not statistically significant.

Megan Ritson[1], Dong Xia[1], Caroline Wheeler-Jones[1] and Helen B. Stolp[1]*

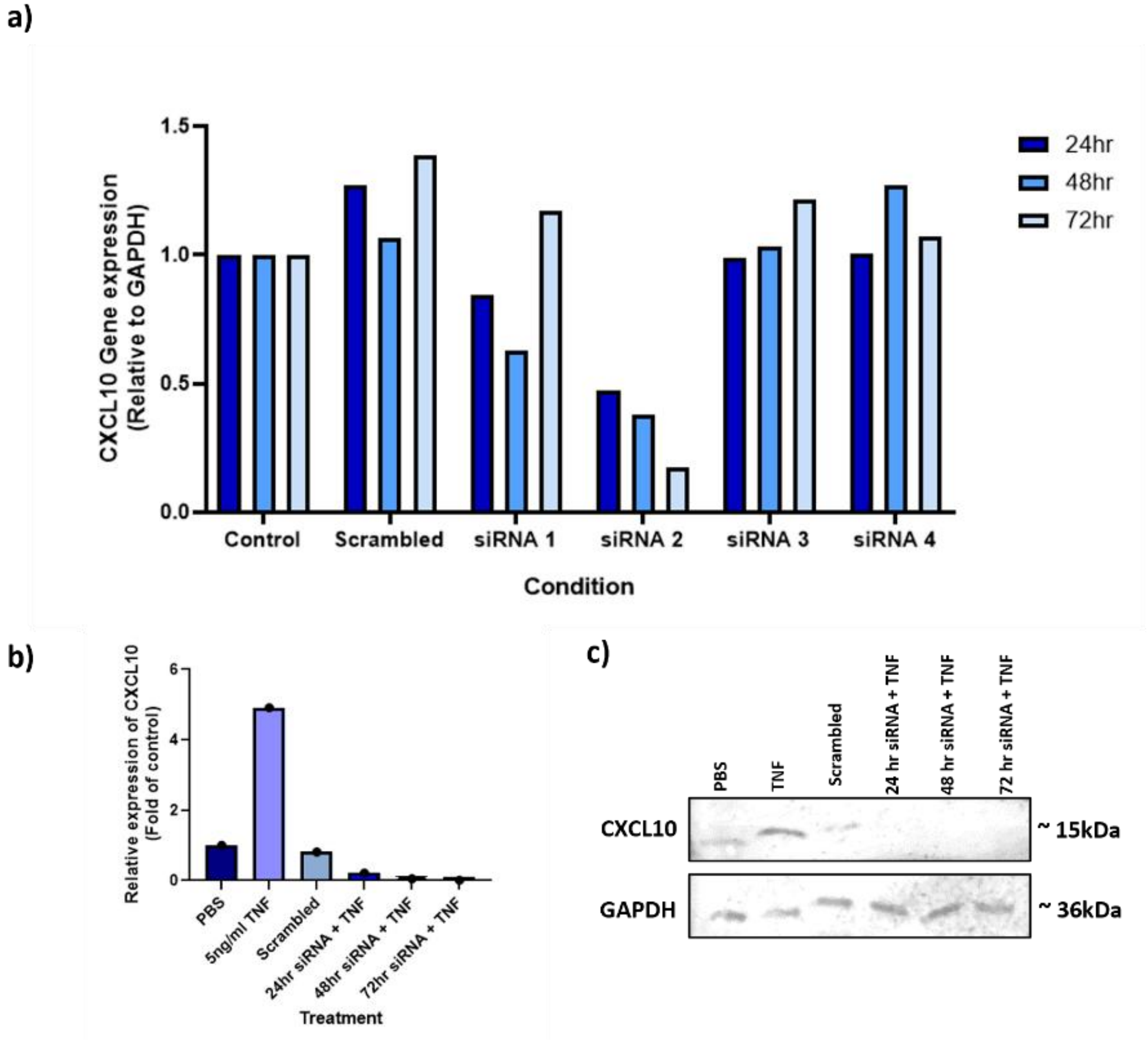

Supplementary Figure 4. Validation of siRNA-mediated CXCL10 knockdown

a) Unstimulated bEnd.3 cells were treated with either vehicle (PBS), scrambled non-targeting small interfering RNA (siRNA), or one of three CXCL10 siRNAs (n=1). The expression of C-X-C Motif Chemokine Ligand 10 *(Cxcl10)* mRNA was quantified at 24, 48 and 72 hours. **b)** *CXCL10* gene expression was quantified following treatment of cells with siRNA 2 and stimulated with 5ng/ml Tumor necrosis factor (TNF)**. c)** Protein expression of CXCL10 was quantified at these same time points to confirm knockdown of the CXCL10 protein (n=1).

Repeated low-level inflammatory challenge leads to alterations in the TNF-CXCL10 signalling pathway in mouse cerebral endothelial cells *in vitro*

Megan Ritson[1], Dong Xia[1], Caroline Wheeler-Jones[1] and Helen B. Stolp[1]*

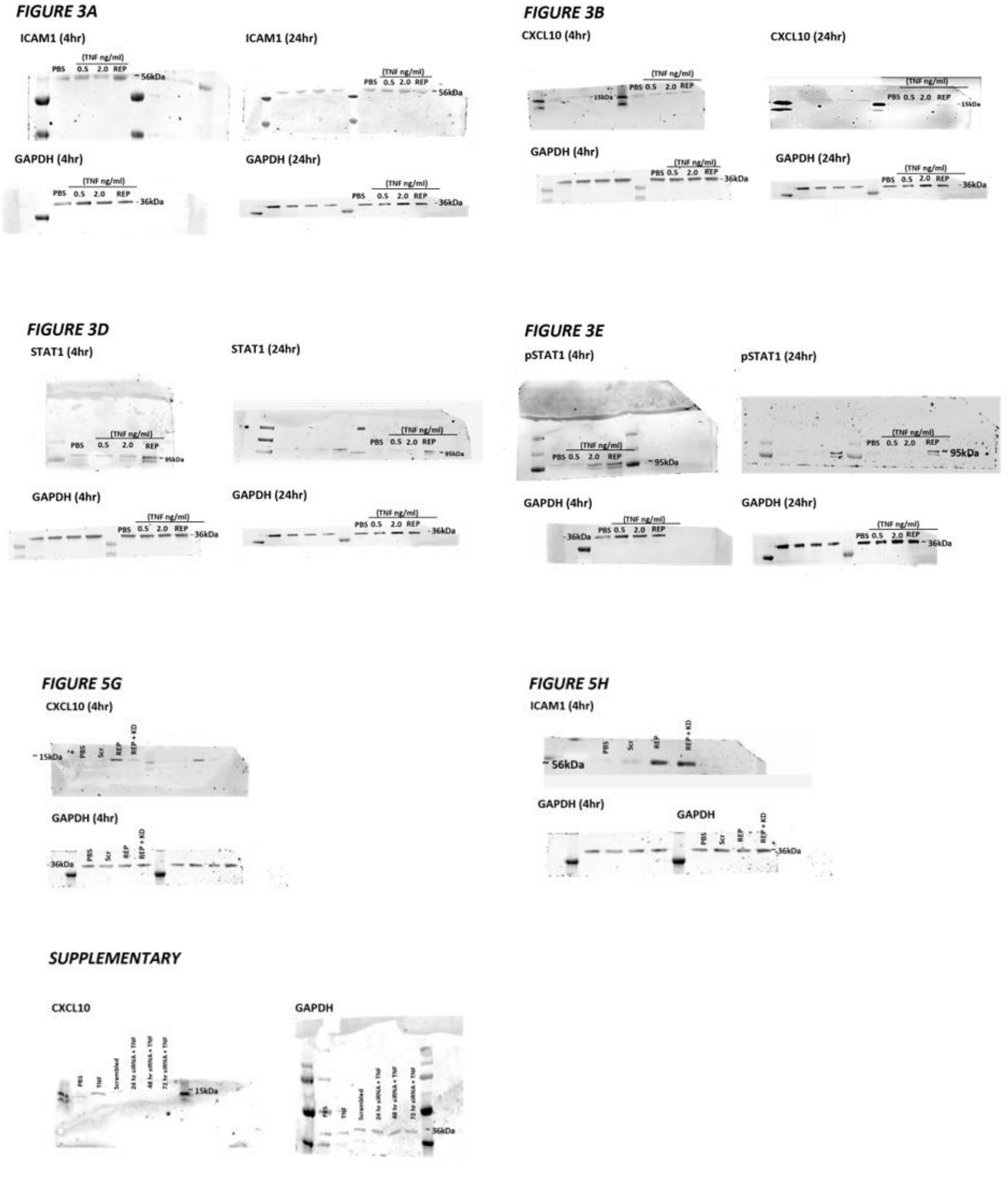

Supplementary Figure 5. Raw western blot images.
