# Supplementary material for: Repeated Low‐Level Inflammatory Challenge Leads to Alterations in the TNF‐CXCL10 Signalling Pathway in Mouse Cerebral Endothelial Cells In Vitro
Source: J Neurochem. 2025 Jun 16;169(6):e70130. doi: 10.1111/jnc.70130 (PMC12169089; doi:10.1111/jnc.70130)
Supplement: Supplementary file 2 — Data S2. [file JNC-169-0-s001.docx]

**Supplementary Data**

**Supplementary Table 1: RNA-Seq datasets used for analysis**

| **Study** | **Species** | **Cell type** | **Injury stimulus** | **Comparisons of interest** | **Control** |
| --- | --- | --- | --- | --- | --- |
| Munji et al, 2019 | *Mouse* | Enriched brain endothelial cells  Derived from VE-Cadherin-Cre_ERT2_ mice. | Middle Cerebral Artery Occlusion (MCAO) – Acute stroke model | Acute stroke vs control, tissues harvested at 24hrs. | Enriched brain endothelial cells from untreated mice |
| Kodali et al, 2020 | *Mouse* | Cerebral endothelial cells  Derived from 8–12-week-old male and female C57BL/6 mice. | Lipopolysaccharide  *(Escherichia coli* O55:B5)  10mg/kg | LPS 15min vs control  LPS 30min vs control  LPS 4hr vs control | Cerebral endothelial cells from PBS injected mice |
| Jambusaria et al, 2020 | *Mouse* | Cerebral endothelial cells  Derived from RiboTag^EC^ (*Cdh5^CreERT2/+^; Rpl22^HA/+^*) mice. | Lipopolysaccharide  *(Escherichia coli* O55:B5)  10mg/kg | LPS 6hr vs control  LPS 24hr vs control | Cerebral endothelial cells from PBS injected mice |

**Supplementary Table 2: Mouse CXCL10 siRNAs**

| **siRNA Mouse CXCL10** | **Target sequence** | **Nanomoles** | **Micrograms** |
| --- | --- | --- | --- |
| **siRNA 1**  **D-042605-01** | CCCAAGUGCUGCCGUCAUU | 2.0 | 26.8 |
| **siRNA 2**  **D-042605-03** | AGAGAUGUCUGAAUCCGGA | 2.0 | 26.8 |
| **siRNA 3**  **D-042605-03** | CCAUAGGGAAGCUUGAAAU | 2.0 | 26.7 |
| **siRNA 4**  **D-042605-04** | CCAUAUCGAUGACGGGCCA | 2.0 | 26.8 |

**Supplementary Table 3: TaqMan primers used for qPCR analysis**

| **Gene name** | **TaqMan accession** | **Probe location (exon boundary)** |
| --- | --- | --- |
| *GAPDH* | Mm99999915_g1 | 2-3 |
| *TNF* | Mm00443258_m1 | 1-2 |
| *CXCL10* | Mm00445235_m1 | 1-2 |
| *IFNG* | Mm01168134_m1 | 3-4 |
| *TRAF2* | Mm00801978_m1 | 4-5 |
| *CXCR3* | Mm99999054_s1 | 2 |
| *ICAM1* | Mm00516023_m1 | 2-3 |

**Supplementary Table 4. Secondary antibodies used for western blotting**

| **IRDye 800CW** | **Supplier** | **Concentration** |
| --- | --- | --- |
| **Goat anti-mouse** | Li-Cor; 926-32210 | 1:10,000 |
| **Goat anti-rabbit** | Li-Cor; 926-32211 | 1:10,000 |
| **Donkey anti-goat** | Li-Cor; 926-32214 | 1:10,000 |

**Supplementary Table 5: Genes showing transcriptomic alterations in 5 or more comparisons**

| **Gene name** | **Gene symbol** |
| --- | --- |
| **C-X-C motif chemokine ligand 10** | *CXCL10* |
| **Early Growth Response 1** | *EGR1* |
| **Ras Homolog Family Member B** | *RHOB* |
| **BTG Anti-Proliferation Factor 2** | *BTG2* |
| **ZFP36 Ring Finger Protein** | *ZFP36* |
| **KLF Transcription Factor 4** | *KLF4* |
| **MAF BZIP Transcription Factor F** | *MAFF* |
| **Immediate Early Response 2** | *IER2* |
| **KLF Transcription Factor 2** | *KLF2* |
| **6-Phosphofructo-2-Kinase/Fructose-2,6-Biphosphatase 3** | *PFKFB3* |
| **TSC22 Domain Family Member 1** | *TSC22D1* |
| **Interferon Regulatory Factor 1** | *IRF1* |
| **TNF Alpha Induced Protein 2** | *TNFAIP2* |
| **CCAAT Enhancer Binding Protein Delta** | *CEBPD* |
| **Pleckstrin** | *PLEK* |
| **Interleukin 23 Subunit Alpha** | *IL23A* |
| **Colony Stimulating Factor 1** | *CSF1* |
| **Immediate Early Response 3** | *IER3* |
| **Nuclear Factor Kappa B Subunit 2** | *NFKB2* |
| **JunB Proto-Oncogene, AP-1 Transcription Factor Subunit** | *JUNB* |
| **Mitogen-Activated Protein Kinase Kinase Kinase 8** | *MAP3K8* |
| **Tubulin Beta 6 Class V** | *TUBB6* |
